# Basic Study for Vaccine Development Targeting Virus Infections

**DOI:** 10.3390/v14010057

**Published:** 2021-12-30

**Authors:** Kyoko Tsukiyama-Kohara, Michinori Kohara

**Affiliations:** 1 Transboundary Animal Diseases Centre, Joint Faculty of Veterinary Medicine, Kagoshima University, Kagoshima 890-0065, Japan; 2 Department of Microbiology and Cell Biology, Tokyo Metropolitan Institute of Medical Science, Tokyo 156-8506, Japan

We acknowledge the publications for this Special Issue, “Basic Studies for Vaccine Development Targeting Virus Infections”.

Three articles and two reviews are published in this Special Issue, and their contents are as follows.

Firstly, “The Mink Circovirus Capsid Subunit Expressed by Recombinant Baculovirus Protects Minks against Refractory Diarrhea in Field”, by Wang et al. They aimed to develop a subunit vaccine against mink circovirus (MiCV). The field trial indicated that in total, only 1.8% of the minks developed typical diarrhea in the vaccinated group compared with 74.5% in the control group. The vaccination could significantly reduce the infection rate of MiCV among the mink herds and could restrain the virus’ shedding from feces. Furthermore, the vaccinated group had a higher average litter size in the following year compared with the control group. Collectively, the results indicate that the subunit vaccine based on the capsid protein can provide reliable protection against MiCV infection.

Secondly, a review article, titled “Tree Shrew as an Emerging Small Animal Model for Human Viral Infection: A Recent Overview”, by Kayesh et al. Tupaia was developed as a small animal model to characterize viral infection, including hepatitis viruses, respiratory viruses, arboviruses and others. They summarized updates regarding human viral infection in the tree shrew model, which highlights the potential of the tree shrew to be utilized for human viral infection and pathogenesis studies.

Thirdly, an article titled “Transcriptomic Characterization Reveals Attributes of High Influenza Virus Productivity in MDCK Cells”, by Ye et al. The authors characterized factors affecting virus productivity by isolating influenza virus A (IAV) subline H1. The authors postulate that the high productivity of IAV hinges on the balance between the suppression of host functions to divert cellular resources and the sustaining of sufficient activities for virus replication.

Fourthly, an article titled “Efficient Control of Zika Virus Infection Induced by a Non-Replicating Adenovector Encoding Zika Virus NS1/NS2 Antigens Fused to the MHC Class II-Associated Invariant Chain”, by Nazerai et al. The authors developed adenoviral vectors encoding the ZIKV non-structural proteins 1 and 2 (NS1/NS2) and employed the strategy of linking the antigens to the MHC-II-associated invariant chain (li) to improve immunogenicity.

Finally, a review article titled “An Overview of Recent Insights into the Response of TLR to SARS-CoV-2 Infection and the Potential of TLR Agonists as SARS-CoV-2 Vaccine Adjuvants”, by Kayesh et al. The authors discuss the recent progress in our understanding of host innate immune responses in SARS-CoV-2 infection, with particular focus on the TLR response. In addition, they discuss the use of TLR agonists as vaccine adjuvants in enhancing the efficacy of COVID-19 vaccines.

Thus, this Special Issue may provide some valuable insights regarding vaccine development for viral infections.

